# Encapsulation of human limbus-derived stromal/mesenchymal stem cells for biological preservation and transportation in extreme Indian conditions for clinical use

**DOI:** 10.1038/s41598-019-53315-x

**Published:** 2019-11-18

**Authors:** Mukesh Damala, Stephen Swioklo, Madhuri A. Koduri, Noopur S. Mitragotri, Sayan Basu, Che J. Connon, Vivek Singh

**Affiliations:** 1 Prof. Brien Holden Eye Research Centre, LV Prasad Eye Institute, Hyderabad, Telangana India; 2 School of Life Sciences, University of Hyderabad, Hyderabad, Telangana India; 3 Atelerix Ltd., Biomedicine West, International Centre for Life, Newcastle Upon Tyne, UK; 4 Institute of Genetic Medicine, Faculty of Medical Sciences, Newcastle University, Newcastle Upon Tyne, UK; 5 Center for Ocular Regeneration (CORE), LV Prasad Eye Institute, Hyderabad, Telangana India

**Keywords:** Mesenchymal stem cells, Translational research

## Abstract

Human limbus-derived stromal/mesenchymal stem cells (hLMSC) can be one of the alternatives for the treatment of corneal scars. However, reliable methods of storing and transporting hLMSC remains a serious translational bottleneck. This study aimed to address these limitations by encapsulating hLMSC in alginate beads. Encapsulated hLMSC were kept in transit in a temperature-conditioned container at room temperature (RT) or stored at 4 °C for 3–5 days, which is the likely duration for transporting cells from bench-to-bedside. Non-encapsulated cells were used as controls. Post-storage, hLMSC were released from encapsulation, and viability-assessed cells were plated. After 48 and 96-hours in culture the survival, gene-expression and phenotypic characteristics of hLMSC were assessed. During transit, the container maintained an average temperature of 18.6 ± 1.8 °C, while the average ambient temperature was 31.4 ± 1.2 °C (*p* = *0.001)*. Encapsulated hLMSC under transit at RT were recovered with a higher viability (82.5 ± 0.9% and 76.9 ± 1.9%) after 3 (*p* = *0.0008)* and 5-day storage (*p* = *0.0104*) respectively as compared to 4 °C (65.2 ± 1.2% and 64.5 ± 0.8% respectively). Cells at RT also showed a trend towards greater survival-rates when cultured (74.3 ± 2.9% and 67.7 ± 9.8%) than cells stored at 4 °C (54.8 ± 9.04% and 52.4 ± 8.1%) after 3 and 5-days storage (*p* > *0.2*). Non-encapsulated cells had negligible viability at RT and 4 °C. Encapsulated hLMSC (RT and 4 °C) maintained their characteristic phenotype (ABCG2, Pax6, CD90, p63-α, CD45, CD73, CD105, Vimentin and Collagen III). The findings of this study suggest that alginate encapsulation is an effective method of hLMSC preservation offering high cell viability over prolonged durations in transit at RT, therefore, potentially expanding the scope of cell-based therapy for corneal blindness.

## Introduction

Loss of corneal stromal transparency is a leading cause of blindness and visual impairment impacting millions of individuals globally^1^. The standard treatment for blinding corneal pathologies is corneal transplantation which suffers from several limitations, including global lack of donor tissue, risk of immune rejection, need for long-term follow-up and compliance with life-long medications^2^. Recent progress in regenerative medicine has provided the opportunity of using mesenchymal stem cells (MSC) for treating corneal pathologies. Pre-clinical studies and early clinical trials using MSC from various sources including human limbus-derived stromal/mesenchymal stem cells (hLMSC) have demonstrated a beneficial therapeutic effect in ameliorating corneal opacification^3–5^. However, safe and reliable methods of storage and transportation of cells for prolonged periods and over long distances, still remain an unmet translational roadblock.

The efficient shipping of cells from production facility to the site of application, while preserving the viability and quality of the cells, is very crucial^6^. Current methods of cryopreservation involve chemicals like dimethyl sulfoxide which itself is harmful to cells. Cryopreserving cells, in addition to being cost ineffective, has the drawbacks of decreased cell-viability^7^, impaired post-thaw function and reduced immunomodulatory properties^8^. The logistical complexity of transporting cells in their frozen state, accompanied by potential loss of function when used directly from the thaw, impedes the accessibility of cells for therapy at remote and rural sites. With increased regenerative research and the increased number of clinical trials, efficient transport (3–5 days in the current global scenario) of stem/progenitor cells from one institution to another where there is no GMP facility, is required. Autologous cells are an option but where autologous cells are not available, as in cases of bilateral eye damage/injury, allogeneic cells may be required. Cells, in general are transported using dry ice or liquid nitrogen modes, which is not cost effective, requires expedited shipping and packaging, suitable infrastructure, and specialised training for thawing and administration. In adverse events like a transportation delay, or change in temperature, cells can thaw and become unusable, or undergo stress affecting their viability and characteristic properties of cells^9^. One of the widely practiced alternatives to prevent this loss is encapsulating the cells in a biological matrix. Hypothermic preservation of encapsulated cells where cells are held in a state of suspended animation at temperatures below the normothermic range of 32 °C-37 °C also combats many of the issues associated with methods like cryopreservation^10^. Alginate is a natural polysaccharide exhibiting excellent biocompatibility and a popularly employed polymer for cell encapsulation. Alginate encapsulation has been reported to show more functionally robust spermatozoa^11^ and oocytes^12^ and to retain the morphological differentiation and adhesion abilities of the Neuroblastoma cells^13^. Recent studies have shown that encapsulating MSCs in alginate hydrogels could be a solution for problems associated with hypothermic storage through extending their preservation shelf life^10,14,15^. However, the reliability of alginate encapsulation has not been previously tested in geographies with high ambient temperatures or after long-distance transportation. This study aimed to test the reliability of alginate encapsulation for storing and transporting hLSMC at room temperature (RT) in temperate climatic conditions.

## Methods and Materials

### Study protocol and donor corneas

This study protocol was approved by the Institutional Review Board, LV Prasad Eye Institute, Hyderabad, India (LEC 05-18-081). Therapeutically accepted and serologically tested cadaveric donor corneas were obtained from Ramayamma International Eye Bank, LV Prasad Eye Institute, Hyderabad, India (http://www.lvpei.org/services/eyebank). Informed consent for using cadaveric corneas was obtained from the donors’ next kin, by the Ramayamma International Eye Bank from where the cadaveric tissues were obtained. Experiments on the human tissue adhered to the declaration of Helsinki. All the experiments in the methodology were performed in triplicates.

### Validating the insulated container for maintenance of hypothermic temperature

To have a reliable system that maintains a normalized range of temperatures irrespective of the extreme atmospheric temperatures, an insulated container with cooling packs (Polybox 7, Softbox Systems, India), pre-conditioned to maintain hypothermic temperatures of ≤30 °C, was assessed (Supplementary Fig. S1). This assessment was done over a duration of 3–5 days, considering it the likely duration taken to transport cells. The internal temperature of the container and the ambient (atmospheric) temperature was recorded every 4 hours during this period.

### Cell culture

The donor corneas were washed with 2% [vol/vol] Antibiotic-Antimycotic (15240062, Thermo Fisher, USA) in Phosphate Buffer Saline (PBS) (14190250, Thermo Fisher, USA) for 2 minutes, followed by another wash with PBS. Iris and endothelial layer were removed for better visibility of the limbus. Complete 360° limbal rims were isolated using a surgical blade in buffered saline and fragmented to minute pieces measuring 1–2 mm long. Tissue fragments were minced for 3–5 minutes using small, curved corneal scissors, in DMEM/F12 media alone (BE04-687F/U1, Lonza, Switzerland). The minced limbal tissue was subjected to collagen digestion by adding 200 IU of reconstituted Collagenase-IV (17104019, Thermo Fisher, USA) in 1 mL of DMEM/F12 media. Tissue digestion was carried out by incubating the limbal tissue for 16 hours at 37 °C with 5% CO_2_ in a humidified incubator.

Post 16-hour incubation, the enzymatic digestion was ceased by adding 2 mL of DMEM/F12 fortified with 2% fetal bovine serum (16000036, Thermo Fisher, USA). The enzyme-digested tissue fragments were washed and sedimented twice at 1000 rpm for 3 minutes, at room temperature (RT) in saline. 3 mL of complete media comprising of DMEM/F12 media fortified with 2% FBS, 1% [vol/vol] Antibiotic-Antimycotic, 10 ng/mL epidermal growth factor (PHG0311L, Thermo Fisher, USA) and 5 µg/mL insulin (12585014, Thermo Fisher, USA) was added to the pellet and kept in culture with culture medium being replaced every 2 days. Pure cultures of hLSMCs were obtained by subculturing. Subculturing was done upon 80–90% confluency. Passage 3 cells were used for all experiments post-quantification for viability using 0.4% Trypan Blue (15250061, Thermo Fisher, USA).

### Encapsulation of hLSMCs

**Figure 1:**
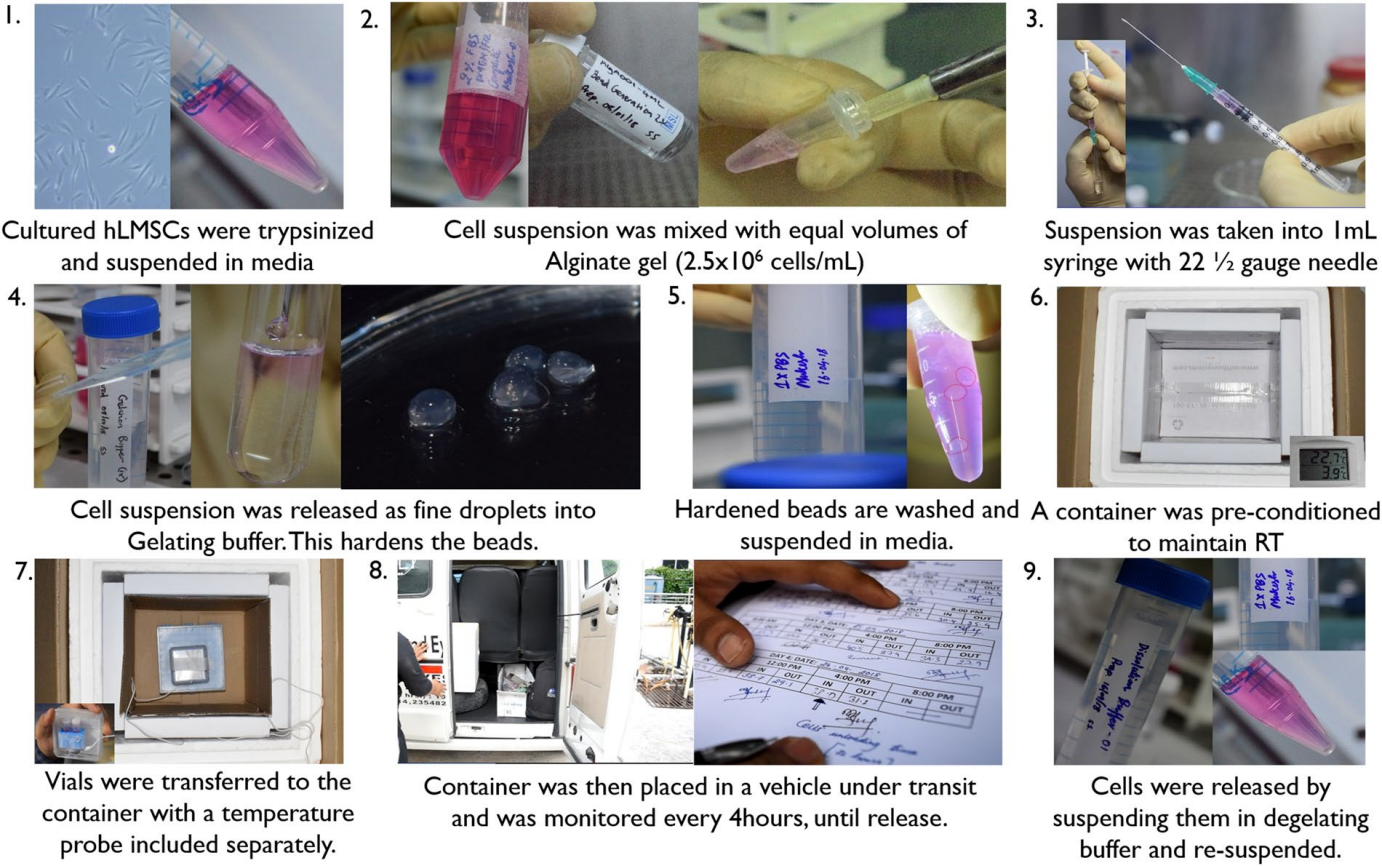
Process of encapsulation and transportation. Schematic diagram of events explaining the encapsulation and transportation of hLMSCs: Alginate-encapsulated hLMSCs, in the form of beads were transported for ~528.67 (±64.2) KMs in real-time conditions for 3–5 days, in a pre-conditioned container.

A cell suspension of hLSMCs harvested from culture was mixed with sodium alginate solution supplied with BeadReady kit commercially available from Atelerix Ltd (UK) at a density of 2.5 × 10^6^ cells/mL. The alginate-cell suspension concoction was slowly dropped into the calcium-chloride based gelation buffer (BeadReady kit) through a 21 1/2 G needle. These droplets of alginate-cell suspension concoction were allowed to stabilize for 8 minutes in the gelation buffer, making the beads polymerize and gelate (Fig. 1). Polymerized beads were washed with complete media and resuspended in 1 mL of fresh complete media.

### Storage and transportation of the encapsulated hLSMCs

Vials with alginate-encapsulated cells in the form of polymerized beads were either refrigerated (4 °C; n = 5) or were kept under transit at RT (n = 5). The internal temperature of the container and the ambient (external) temperature was recorded every 4 hours, until 3–5 days (Fig. 1). The encapsulated cells were transported between three towns around Hyderabad, with a transport distance of ~528.67 (±64.2) kms. The vehicle used for the transit was a standard carrier vehicle. The external temperature outside the container was considered as control temperature. An equal number of the non-encapsulated cells were either stored or transported along, as above. All the packaging was done in controlled conditions. This experiment was performed in triplicates.

### Release of the hLSMCs from encapsulation

Post transit, alginate beads encapsulating the cells were washed with PBS. They were added to 1.3 mL of dissolution buffer (trisodium citrate based), supplied with BeadReady kit and allowed to dissolve for 5 minutes with gentle agitation releasing the cells from the alginate beads. Cells suspended in the dissolution buffer were sedimented by centrifugation at 1500 rpm for 5 minutes. The sedimented cell pellet was resuspended in complete medium.

### Quantifying the viable cells recovered

The number of viable (unstained) cells recovered from each vial that were either stored at 4 °C or transported at RT was quantified using 0.4% Trypan blue solution and counted using a hemocytometer. Post quantification, the cells from the vials of same storage conditions (n = 5, each of RT and 4 °C), were pooled together. Pooled cells, along with non-encapsulated cells (cultured under standard culture conditions) as a control, were plated in equal numbers for further analysis of determining their relative survival, gene expression, and phenotypic biomarkers expression.

### Determining the relative rate of survival using MTT assay

Post-release from 3-day and 5-day storage or transit, and their quantification for viability, the cells were plated in triplicates in a 12-well plate, at a density of 20,000 cells/cm^2^ and cultured for 48 and 96 hours at 37 °C with 5% CO_2_ in a humidified incubator. The relative survival rates of the cells against the control of non-encapsulated (cultured under standard culture conditions) cells were assessed using MTT reagent (M6494, Thermo Fisher, USA). Each well was added with 200 µL of 0.25 mg/mL MTT reagent in culture medium devoid of FBS and incubated for 1 hour at 37 °C in 5% CO_2_ chamber. The formazon crystals were solubilized in 200 µL of Dimethyl Sulfoxide (D2650, Sigma Aldrich, USA) for 5 minutes at 37 °C in 5% CO_2_ chamber. The concentration was determined by reading the absorbance in duplicates at 570 nm using a spectrophotometer, against a blank.

### Assessment of the phenotypic marker expression

Encapsulated cells that were either transported at RT or were under storage at 4 °C for 3–5 days were released and quantified for viability. Cells were cultured on coverslips in 12-well culture plates at a density of 20,000 cells/cm^2^ at 37 °C with 5% CO_2_ in a humidified incubator for 48 hours. These cells were assessed for the expression of characteristic biomarkers of the hLMSC phenotype. Cultured cells were washed with PBS and fixed using 4% paraformaldehyde in PBS, for 20 minutes, followed by a 10-minute wash in PBS, twice. The cells were permeabilized using 0.03% [vol/vol] Triton-X in PBS, followed by two 5-minute washes in PBS. Cells were incubated for 1 hour with 2.5% BSA in PBS, to block the non-specific protein-protein interactions. All the incubations were carried out at RT in moist conditions. The blocking solution was removed and cells were incubated for 2 hours with primary antibodies in 100 µL of 1% BSA in PBS. The antibody panel was composed of (a) ABCG2 (1:100, 18841, Santa Cruz Biotechnology, USA), Pax6 (1:300, 901301, BioLegend, USA), p63-α (1:100, 4892S, Cell Signalling technology, USA) and Col-III (1:100, ab7778, Abcam, UK), as positive markers of the human limbal stem cell phenotype; HLA-DR (1:100, ab55152, Abcam, UK), and CD45 (1:100, 13197, Cell Signalling Technology, USA) as negative marker for mesenchymal origin, (b) CD73 (1:100, 13160, Cell Signalling Technology, USA), CD105 (1:100, 376381, Santa Cruz Biotechnology, USA), and VIM (1:100, 6260, Santa Cruz Biotechnology, USA) as positive markers of the mesenchymal phenotype. The p63-α antibody used in our study recognizes both ∆Np63-α and TAp63-α components (https://media.cellsignal.com/pdf/4892.pdf). Cells on coverslips were washed twice in PBS for 5 minutes each, after the incubation with primary antibodies. Cells were then incubated for 45 minutes in 100 µL with secondary antibodies (1:400) of 1% BSA in PBS, followed by three 10-minute washes in PBS. This antibody panel was defined abiding by the International Society for Cellular Therapy’s guidelines of minimal criteria for defining multipotent mesenchymal stromal cells^16^. The panel of secondary antibodies included anti-mouse Alexa Fluor 488 (A11001, Thermo Fisher, USA) and anti-rabbit Alexa Fluor 488 (A11008, Thermo Fisher, USA). Cells were mounted using Fluorosheild mounting medium with DAPI (ab104139, Abcam, UK) and imaging was done using a fluorescent microscope (Axio Scope A1, Carl Zeiss AG, Germany) with 20x–40x objective. This experiment was repeated thrice. The number of cells positive for a given biomarker is expressed in the form of percentage by analysing the images captured from the central (1 image) and peripheral areas (2 images) of the coverslip. It is represented in the tabular format for better understanding. The lack of expression is denoted by (−) and <25% of cells showing positive expression is denoted by (++), 25–50% is denoted by (+++) and >90% cells being positive is denoted as (++++).

### Quantification of the gene expression using real-time PCR

One million cells of each storage category after their release from encapsulation were used for quantifying the gene expression. Freshly lysed *trypsinized* cells from the culture were used as the control. Total RNA was isolated using Trizol (15596018, Thermo Fisher, USA) method and converted to cDNA using the Superscript-III (1808051, Thermo Fisher, USA) at 1 µg/µL of RNA per 20 µL reaction mix. The synthesized cDNA was subjected to real-time PCR, using Maxima SYBR Green kit (K0221, Thermo Fisher, USA) with 200 ng template per 25 µL reaction mix. The reaction was carried out in a detection system (Applied Biosystems, USA). Reactions were run in duplicates. GAPDH was used as a housekeeping gene in these experiments. The gene expression data were normalized to control the variability in expression levels to the geometric mean of the housekeeping gene. The data was analysed using the 2^−ΔΔCT^ method. The primer sequences are listed in the Table 1.

**Table 1:** List of primers and their nucleotide sequences used in this study for the gene expression experiments.

Sl #	Primer	Sequence	Size	T_m_ (°C)
1	GAPDH	Forward: ACCACAGTCCATGCCATCAC	452 bp	55 °C
		Reverse: TCCACCACCCTGTTGCTGTA		
2	CD90	Forward: CGCTCTCCTGCTAACAGTCTT	142 bp	60 °C
		Reverse: CAGGCTGAACTCGTACTGGA		
3	PAX-6	Forward: ATAACCTGCCTATGCAACCC	208 bp	58 °C
		Reverse: GGAACTTGAACTGGAACTGAC		
4	p63-α	Forward: GAGGTTGGGCTGTTCATCAT	183 bp	57 °C
		Reverse: AGGAGATGAGAAGGGGAGGA		

### Statistical analysis

Statistical analyses were done using the GraphPad software (GraphPad Software, San Diego, CA, http://www.graphpad.com). Comparisons were made using Mann-Whitney U test for non-parametric data. The data is presented as mean values ± SD, obtained from 3–10 independent experiments performed. Values of *p* < *0.05* were considered to be significant. *p ≤ *0.05*, **p ≤ *0.001*. Values of *p* > *0.05* were considered insignificant and were represented with #.

## Results

### Maintenance of hypothermic temperatures in the pre-conditioned container

**Figure 2:**
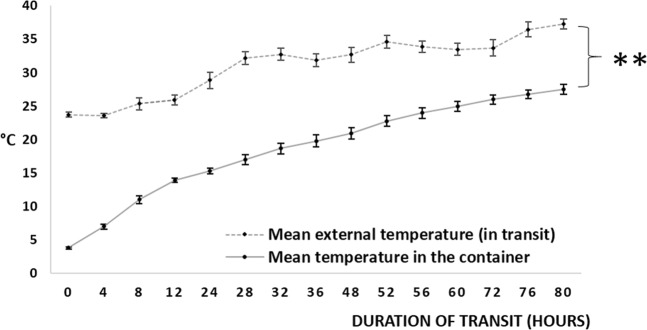
Maintenance of hypothermic temperatures in the pre-conditioned container. Mean external (ambient) and internal temperatures of the conditioned container: A Styrofoam container was conditioned to maintain hypothermic temperatures, by loading with pre-chilled (2–8 °C, 72 hours) gel pads (6 no.s covering all sides of a small box holding vials of cells). This was loaded with alginate beads (without cells) in media, at 13–15 °C of the container’s internal temperature, packed, sealed and kept under transit (n = 10). Temperatures were recorded every four hours, up to 80 hours. **p ≤ *0.001*.

The container maintained an average temperature of 18.62 ± 1.82 °C (range: 13.91 °C to 27.52 °C) where the average ambient temperature was 31.43 ± 1.2 °C (range: 28.85 °C to 38.40 °C) over a duration of 3–5 days (Fig. 2). This experiment was repeated (n = 10) and the data was statistically significant (*p* < *0.0001*). The container had maintained the hypothermic range of temperatures consistently over a period of varying seasons and weather across the year.

### Effect of temperature on viable cell recovery

**Figure 3:**
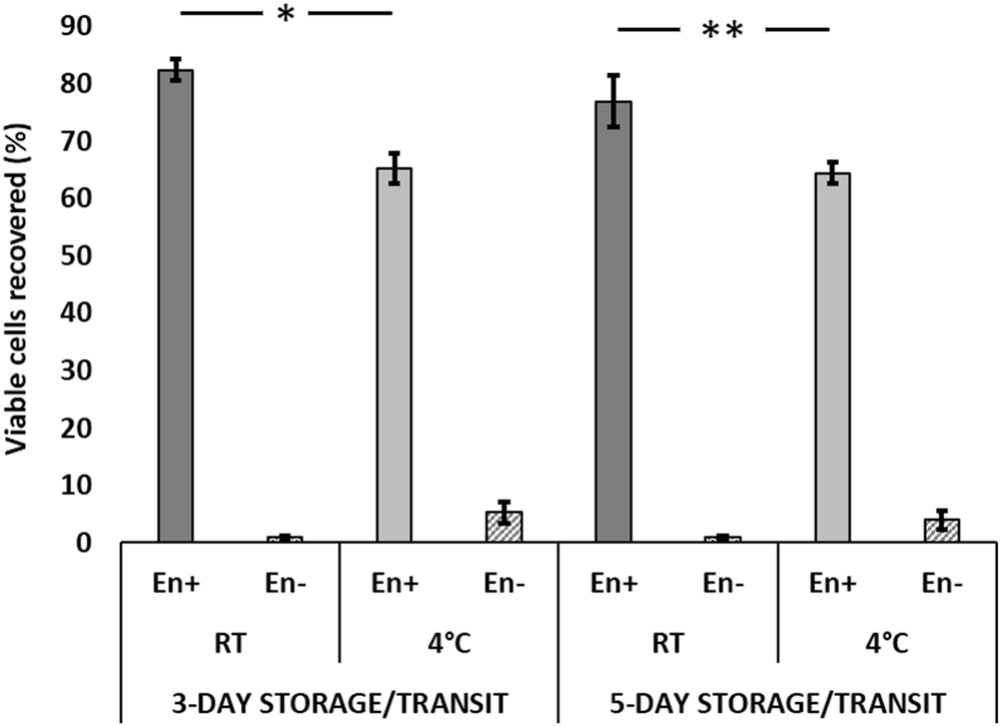
Effect of temperature on viability of encapsulated cells. Mean recovery of the viable encapsulated cells: The storage temperature had an insignificant effect on the viability of encapsulated cells. Encapsulated cells were kept under transit at 4 °C and RT (5 each vials with 0.5 × 10^6^ encapsulated cells/vial) for 3–5 days (n = 3). Cells were released from encapsulation after transit and quantified for viability by dye exclusion method using 0.4% Trypan blue. The average cell viability at given temperature and duration of the storage, was expressed in percentage, with error bars. **En+**: Encapsulated, **En−**: Non-encapsulated. *p = 0.0104, **p = 0.0008.

The temperature in the storage conditions had an insignificant effect on the recovery of viable cells from encapsulation. Encapsulated cells recovered after 3-day transit at RT had an average viable recovery of 82.45 ± 0.87% (n = 3) cells while the cells stored at 4 °C had 65.19 ± 1.19% (n = 3, *p* = *0.0008*) viability. After 5-day transit at RT, encapsulated cells had 76.96 ± 1.98% (n = 3) and cells stored at 4 °C had 64.45 ± 0.81% (n = 3, *p* = *0.0104*) of viable cell recovery (Fig. 3). The non-encapsulated cells stored at RT did not show more than 1% viability during both 3-day and 5-day transit. Non-encapsulated cells stored at 4 °C showed a mean recovery of 5.33% on 3-day storage and up to 4% after 5-day storage.

### Relative survival rate of the encapsulated cells

**Figure 4:**
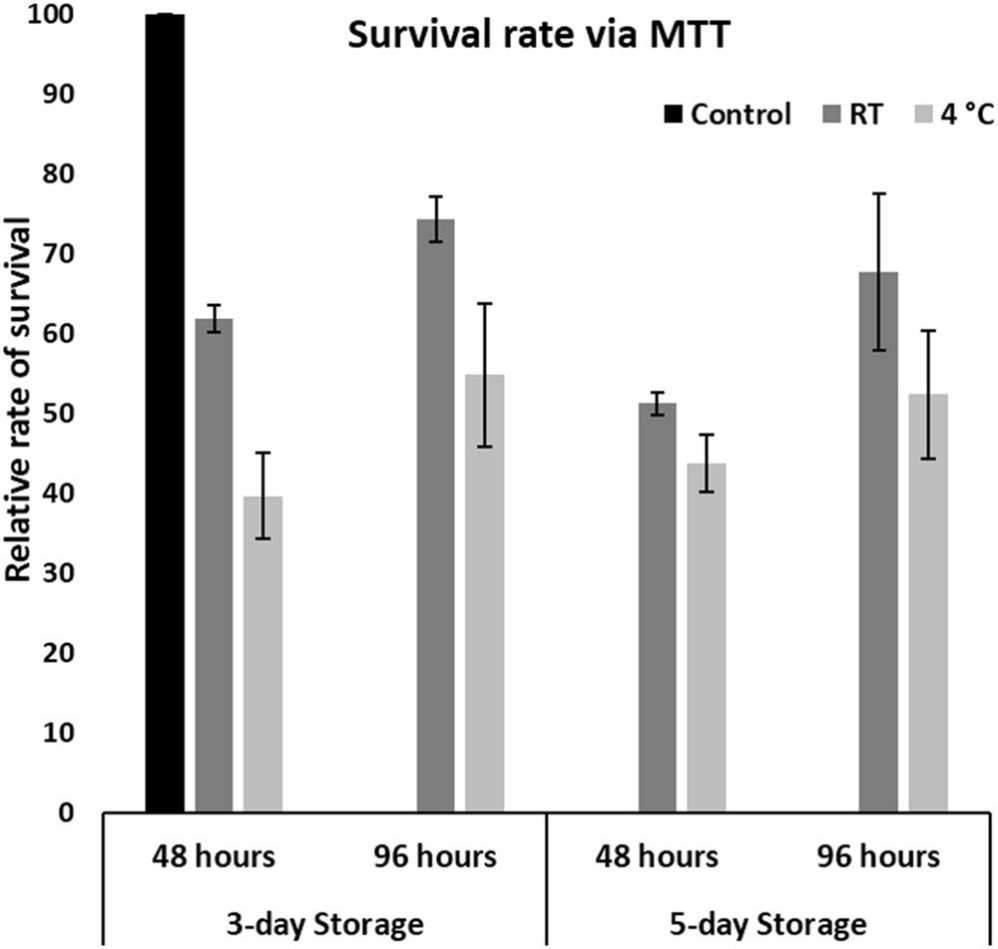
Survival rate of the encapsulated cells. RT-stored encapsulated cells show greater survival than 4 °C-stored encapsulated cells. Alginate encapsulated cells stored/transported at 4 **°**C and RT for 3–5 days (n = 3), were released and plated in triplicates of equal numbers after quantifying for viability. The regular non-encapsulated cells were plated in same number as controls in all the cases. Cells were cultured for 48 hours and 96 hours and assessed for rate of survival using MTT assay. The average rates of survival were expressed in percentage, by comparing absorbance of given category relative to the standard/control group of cells (capped to 100%).

The encapsulated cells kept under transit at RT for 3 days exhibited a relative survival rate of 61.93 ± 1.68% after 48 hours compared to the control group. This increased to 74.34 ± 2.89% in the subsequent 48 hours. Cells that were under transit for 5 days, showed 51.24 ± 1.38% survival after 48 hours that increased to 67.74 ± 9.78% after 96 hours (Fig. 4). On the other hand, the encapsulated cells stored under refrigerated conditions for 3-days, have shown attachment of about 39.67 ± 5.32% after 48 hours and which increased to 54.8 ± 9.04% after 96 hours. Upon 5-day refrigeration, the cell attachment was 43.77 ± 3.53% after 48 hours and 52.35 ± 8.07% after 96 hours.

### Phenotypic expression of the biomarkers

**Figure 5:**
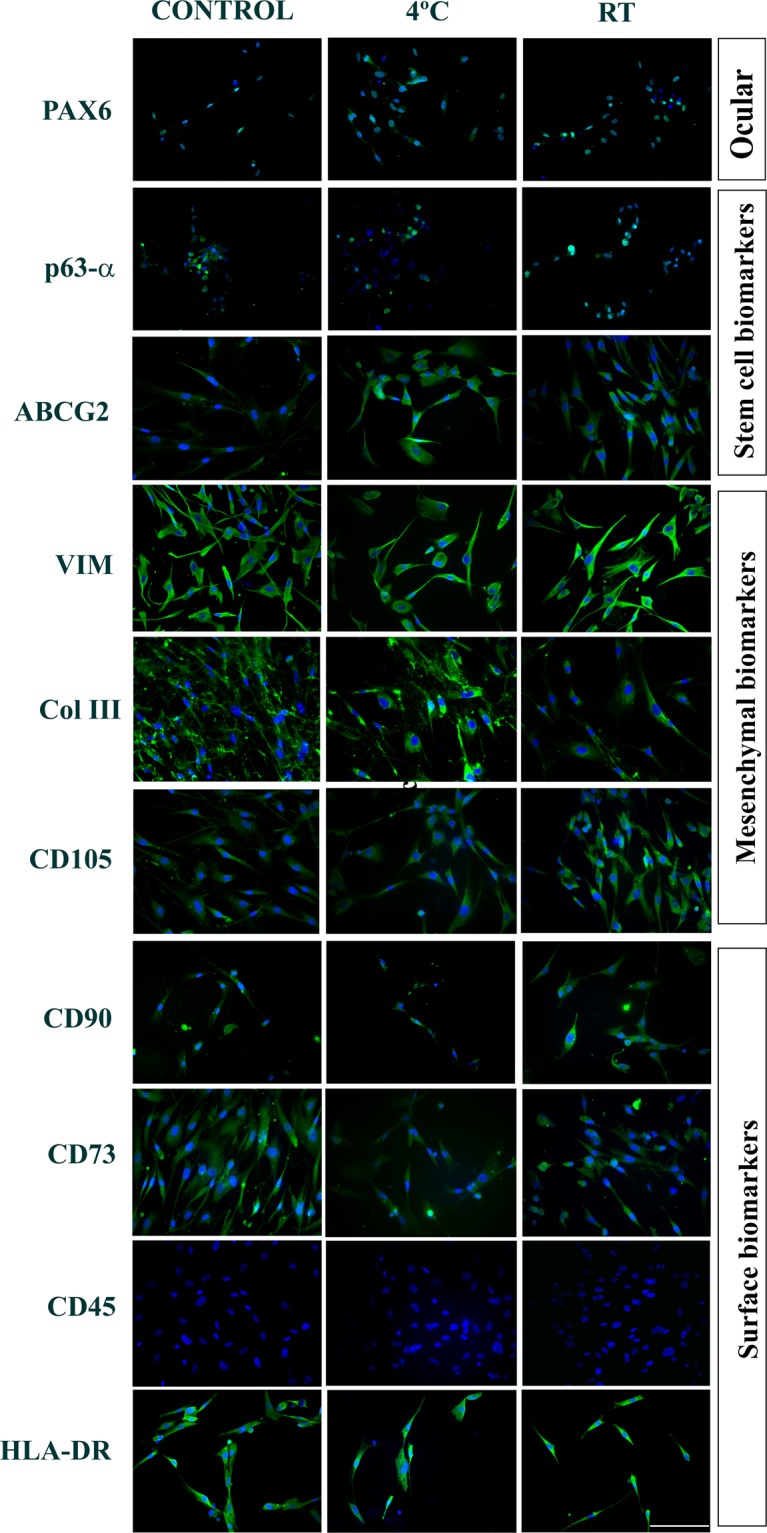
Phenotypic expression of the biomarkers. Immunostaining of the encapsulated hLSMCs under transit for 3 days: Alginate encapsulated hLSMCs of both groups, stored/under transit for 3 days have shown the expression of Pax6^+^, stem-cell biomarkers (ABCG2^+^, p63-α^+^) and the mesenchymal biomarkers (VIM^+^, CD90^+^, CD105^+^ and CD45^−^) with respect to the control cells. ***Blue:*** DAPI, nuclear stain. ***Scale:*** 100 µM.

**Figure 6:**
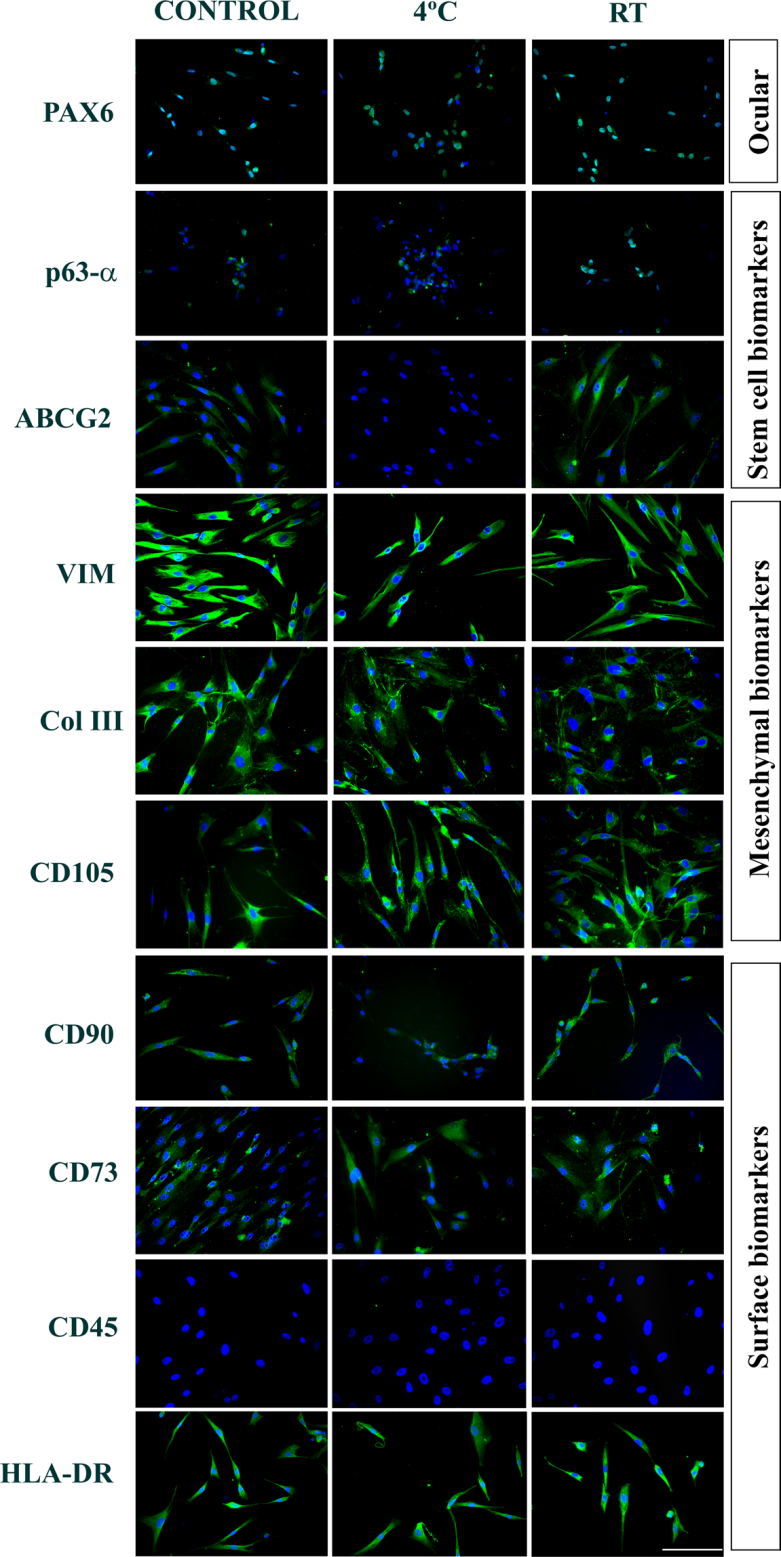
Quantification of the gene expression using real-time PCR. Immunostaining of the encapsulated hLSMCs under transit for 5 days: Alginate encapsulated hLSMCs stored at 4 °C did not show expression of the stem-cell (ABCG2^−^). The RT group cells have showed similar phenotype as the control group (ABCG2^+^, Pax6^+^ p63-α^+^, VIM^+^, CD90^+^, CD105^+^, CD45^−^, HLADR^+^, Col-III^+^, and CD73^+^). ***Blue:*** DAPI, nuclear stain. ***Scale****:* 100 µM.

Encapsulated cells under transit at RT have shown the similar (default) phenotype with the control group of cells, during both 3-day and 5-day transit. Encapsulated cells under storage at 4 °C showed expression of ABCG2 after 3-day storage but not at 5-day refrigeration (Figs 5 and 6). The expression of the rest of the biomarkers by both RT and 4 °C groups was similar to the control cells, showing the positive expression of Pax6^+^ and stem cell markers (p63-α^+^, ABCG2^+^) and the biomarkers for mesenchymal origin (VIM^+^, CD105^+^, CD90^+^, CD45^−^) and the other surface biomarkers Col-III^+^, and CD73^+^. Although HLA-DR is considered negative marker for the mesenchymal origin, we have found this marker to be positively expressing in all the groups of cells irrespective of encapsulation. The number of cells (categorized to %) with positive expression for a given characteristic biomarker is represented in the tabular format (Table 2).

**Table 2:** Tabular format denoting the number of cells showing positive expression of the phenotypic biomarkers.

Type	Biomarker	In transit for 3 days			In transit for 5 days		
		Control	4 °C	RT	Control	4 °C	RT
Ocular	Pax6	++	++	++	++	++	++
Stem Cell	p63-α	+	+	+	+	+	+
	ABCG2	+++	+++	+++	+++	−	+++
Mesenchymal	VIM	++++	++++	++++	++++	++++	++++
	Col III	++++	++++	++++	++++	++++	++++
	CD105	++++	++++	++++	++++	++++	++++
Surface	CD90	++++	++++	++++	++++	++++	++++
	CD73	++++	++++	++++	++++	++++	++++
	CD45	−	−	−	−	−	−
	HLA-DR	++++	++++	++++	++++	++++	++++

(−): No expression; (+): <25% cells are positive, (++): 25–50%, (+++): 50–90%, (++++): >90% cells are positive.

### Quantifying the gene expression (RT-PCR)

**Figure 7:**
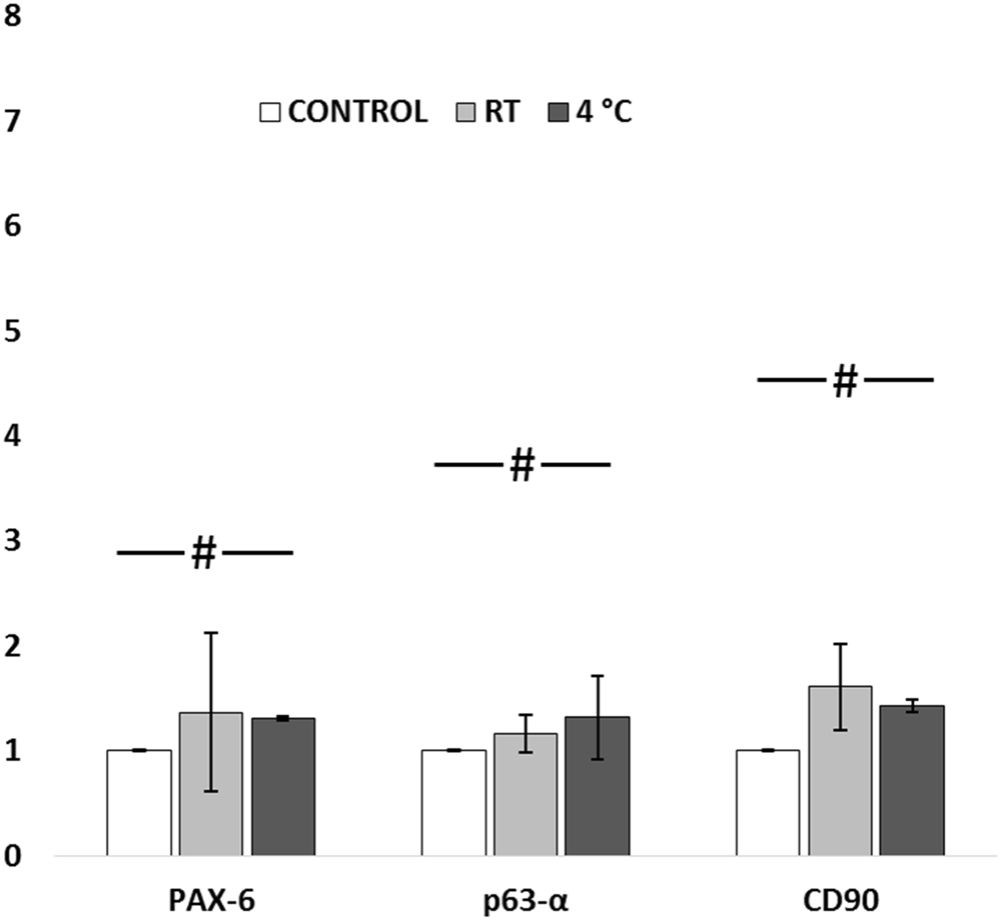
Quantification of gene expression of encapsulated cells, under transit for 3 days: Cells stored at 4 °C have shown 0.6-fold increased expression of ABCG2, PAX-6 and p63-α; ~2-fold increased expression of CD90 when compared to control. Insignificant fold change of expression was found between the control and RT groups for all the three markers. ^#^*p* > *0.11*.

Although encapsulated cells stored at RT and 4 °C showed higher levels of PAX-6, p63-α, and CD90 expression as compared to the control group, these differences were not statistically significant (Fig. 7, *p* > *0.11*).

## Discussion

This study aimed to evaluate the efficacy of alginate encapsulation in maintaining the viability and properties of hLMSC while being stored and transported at RT in a real-life ground-transportation scenario. The study found that while non-encapsulated cells had negligible viability at RT and 4 °C, encapsulated hLMSC (RT and 4 °C) maintained high viability, had good survival in culture and retained adequate phenotype expression. The phenotypic assessment of the encapsulated cells in comparison with control groups showing the number of cells positive for a given biomarker is given in Table 2. A similar trend of the percentage of cells expressing a biomarker was observed. We have found positive expression of HLA-DR in all the groups of cells. Many earlier studies have shown similar findings of the positive expression of HLA-DR in the normal cornea towards periphery and the limbus^17–19^. The findings of this study suggest that alginate encapsulation is an effective method of hLMSC preservation and transport at RT for up to 3 to 5 days, which would allow these cells to be shipped to remote locations and therefore, potentially expand the scope of cell-based therapy for corneal blindness.

Corneal stromal stem cells and more recently hLMSC have been studied for their ability to restore corneal transparency^3^ through corneal stromal regeneration^20^. The therapeutic potential of these cells for treating various corneal pathologies is currently being explored in clinical trials and the initial reports have shown enhancement in visual parameters and corneal epithelization, neovascularization and clarity^4,21,22^. These cells may eventually evolve into a simpler non-invasive alternative to corneal transplantation, thereby reducing the global demand for donor corneas. Further expansion of this therapeutic advancement is hindered by the bottlenecks of lacking proper preservation and transport methods towards the delivery of these cells without affecting their characteristic properties. The maintenance of appropriate temperature is a crucial and integral factor for optimal shelf life of the cells^23^. Despite the ambient temperature fluctuations between 28.9 to 38.4 °C, not only was the insulated container able to maintain significantly lower temperatures of 13.9 to 27.5 °C, but alginate encapsulation also allowed most cells to survive while in transit. The proportion of the encapsulated cells that were lost in the transit, may be considered to have undergone apoptosis. However, without encapsulation almost all cells perished within the same amount of time. Ability to transport cells at RT circumvents the usage of dry ice, which is currently categorized as restricted item for airborne transport (https://www.fedex.com/in/domestic/services/regulatoryguidelines.html) and of any expensive equipment required to maintain chilled temperature during shipping. This would potentially translate into significantly lower costs for cell storage and transportation.

Achieving optimal cell viability and unaffected cell phenotype forms an integral crux of a validated shipping protocol. Similar reports of good viability when stored at room temperatures have been reported earlier with hydrogel encapsulation with^24,25^ or without^23^ extracellular matrix components. However, there are two distinct novelties of this study: (i) previous studies did not test the efficacy of the preservation methods in retaining the properties of stem cells obtained from primary tissues of human origin, while in actual transit.; (ii) while the experimental temperature ranges tested previously were 22–25 °C^16^ or 11–23 °C^10^ in controlled laboratory set-ups, in this study the external ambient temperature ranged from 28.9 to 38.4 °C in real-life conditions. These results imply that it may be possible to send the alginate encapsulated cells to remote locations for their application using ground transportation, which would significantly lower the shipping costs involved. The remote and rural areas, by having equipped with one centrifuge and a pipette, shall be able to release the encapsulated cells, without the necessity of having a cell culture facility. Additionally, all the reagents and procedures employed in the process of cell encapsulation are FDA approved. This would ease the regulatory constraints on the clinical translation and expansion of the technique^26^. However, this study is limited by lacking serum free culture methods and the study of therapeutic properties of the encapsulated cells, which are underway in the further phase of this study.

In conclusion, this study aimed to test the reliability of alginate encapsulation for storing and transporting hLSMC at RT in temperate climatic conditions and the findings of this study suggest that alginate encapsulation is an effective method of hLMSC preservation offering high cell viability over prolonged durations in real-life transit conditions. The simplicity of the encapsulation process combined with the cost-effectiveness of ground-transportation makes alginate encapsulation an attractive option for furthering the scope and scale of cell-based therapy for corneal blindness particularly in the developing world.

## Supplementary information

Supplementary figure

## References

[1] Whitcher JP, Srinivasan M, Upadhyay MP (2001). Corneal blindness: a global perspective. Bull. World Health Organ..

[2] Williams KA (2006). How effective is penetrating corneal transplantation? Factors influencing long-term outcome in multivariate analysis. Transplantation..

[3] Basu S (2014). Human limbal biopsy–derived stromal stem cells prevent corneal scarring. Sci transl med..

[4] Basu S, Damala M, Singh V (2017). Limbal stromal stem cell therapy for acute and chronic superficial corneal pathologies: early clinical outcomes of the Funderburgh technique. Invest. Ophthalmol. & Vis. Sci..

[5] Calonge M (2018). A proof-of-concept clinical trial using mesenchymal stem cells for the treatment of corneal epithelial stem cell deficiency. Transl Res..

[6] Whiteside TL (2011). Shipping of therapeutic somatic cell products. Cytotherapy..

[7] Karlsson JO, Toner M (1996). Long-term storage of tissues by cryopreservation: critical issues. Biomaterials..

[8] Chinnadurai R (2016). Cryopreserved mesenchymal stromal cells are susceptible to T‐cell mediated apoptosis which is partly rescued by IFNγ licensing. Stem Cells..

[9] Lioznov M (2008). Transportation and cryopreservation may impair haematopoietic stem cell function and engraftment of allogeneic PBSCs, but not BM. Bone marrow transplantation.

[10] Swioklo S, Connon CJ (2016). Keeping cells in their place: the future of stem cell encapsulation. Expert. Opin. Biol. Ther..

[11] Faustini M (2010). Boar sperm encapsulation reduces *in vitro* polyspermy. Reproduction in Domestic Animals.

[12] Sakurai T, Kimura M, Sato M (2005). Temporary developmental arrest after storage of fertilized mouse oocytes at 4 C: effects on embryonic development, maternal mRNA processing and cell cycle. Molecular human reproduction.

[13] Tamponnet C (1990). Storage and growth of neuroblastoma cells immobilized in calcium-alginate beads. Applied microbiology and biotechnology.

[14] Swioklo S, Constantinescu A, Connon CJ (2016). Alginate-encapsulation for the improved hypothermic preservation of human adipose-derived stem cells. Stem Cells Transl. Med..

[15] Swioklo S, Ding P, Pacek AW, Connon CJ (2017). Process parameters for the high-scale production of alginate-encapsulated stem cells for storage and distribution throughout the cell therapy supply chain. Process Biochem..

[16] Dominici M (2006). Minimal criteria for defining multipotent mesenchymal stromal cells. The International Society for Cellular Therapy position statement. Cytotherapy..

[17] Treseler PA, Sanfilippo F, Foulks GN (1984). The expression of HLA antigens by cells in the human cornea. Am J Ophthalmol..

[18] Hamrah P, Zhang Q, Liu Y, Dana MR (2002). Novel characterization of MHC class II–negative population of resident corneal Langerhans cell–type dendritic cells. Invest. Ophthalmol. & Vis. Sci..

[19] Hamrah P, Huq SO, Liu Y, Zhang Q, Dana MR (2003). Corneal immunity is mediated by heterogeneous population of antigen‐presenting cells. J Leukoc Biol..

[20] Hassell JR, Birk DE (2010). The molecular basis of corneal transparency. Exp. Eye Res..

[21] Damala, M., Kethiri, A. R., Tavakkoli, F., Raju, E. & Singh. V. The basics of stem cells and their role in vision in *Trends in Life Science**Research* (ed. Sinha P. R., and Umesh P. S.). 97–124 (Nova Science, 2018).

[22] Funderburgh J (2018). Limbal stromal stem cell therapy for acute and chronic superficial corneal pathologies: one-year outcomes. Invest. Ophthalmol. & Vis. Sci..

[23] Olson WC (2011). Shipping blood to a central laboratory in multicenter clinical trials: effect of ambient temperature on specimen temperature, and effects of temperature on mononuclear cell yield, viability and immunologic function. J. Transl. Med..

[24] Wang J (2015). Transporting cells in semi-solid gel condition and at ambient temperature. PLoS One..

[25] Nicodemus GD, Bryant SJ (2008). Cell encapsulation in biodegradable hydrogels for tissue engineering applications. Tissue. Eng. Part B Rev..

[26] Wright B (2012). Enhanced viability of corneal epithelial cells for efficient transport/storage using a structurally modified calcium alginate hydrogel. Regenerative medicine.

