# Supplementary material for: Encapsulation of human limbus-derived stromal/mesenchymal stem cells for biological preservation and transportation in extreme Indian conditions for clinical use
Source: Sci Rep. 2019 Nov 18;9:16950. doi: 10.1038/s41598-019-53315-x (PMC6861256; doi:10.1038/s41598-019-53315-x)
Supplement: Supplementary file 1 — Supplementary figure [file 41598_2019_53315_MOESM1_ESM.docx]

**Encapsulation of human limbus-derived stromal/mesenchymal stem cells for biological preservation and transportation in extreme Indian conditions for clinical use**

Mukesh Damala^1, 2^, Stephen Swioklo^3^, Madhuri A Kondapaka^1^, Noopur S Mitragotri^1^, Sayan Basu^1,5^, Che J Connon ^4^, Vivek Singh^1,5, *^

1 Prof. Brien Holden Eye Research Centre, LV Prasad Eye Institute, Hyderabad, Telangana, India.

2 School of Life Sciences, University of Hyderabad, Hyderabad, Telangana, India.

3 Atelerix Ltd., Biomedicine West, International Centre for Life, Newcastle Upon Tyne, UK.

4 Institute of Genetic Medicine, Faculty of Medical Sciences, Newcastle University, Newcastle Upon Tyne, UK.

5 Center for Ocular Regeneration (CORE), LV Prasad Eye Institute, Hyderabad, Telangana, India.

**Corresponding Author-**

***Vivek Singh,** Brien Holden Eye Research Center, LV Prasad Eye Institute, Hyderabad 500034, India. E-mail: [**viveksingh@lvpei.org**](mailto:viveksingh@lvpei.org)

**Supplementary figure:**

**Figure 1:**

**
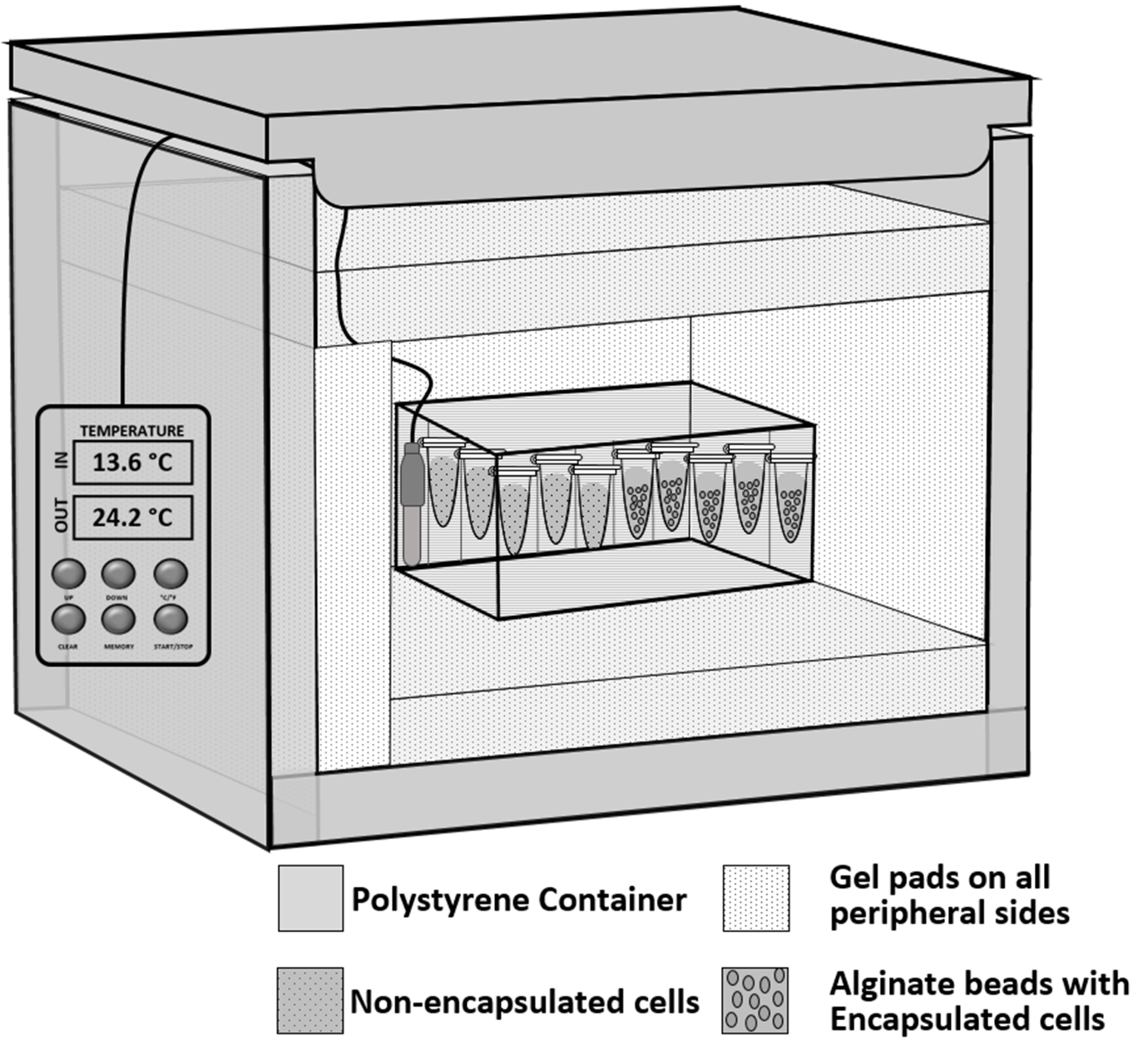
**

**Schematic diagram of the container for transport at hypothermic temperatures:**

A pre-conditioned styrofoam container that maintains hypothermic temperatures of <30°C. Vials holding the cells were surrounded by gel packs on all peripheral sides, pre-chilled at 4°C (72hours). This was equipped with a digital thermometer than measures both internal (via device’s probe) and external temperatures. Container is sealed after loading vials and was kept under transit for upto 5days.
